# Comparison of Lung-RADS Version 2022 and British Thoracic Society Guidelines in Classifying Solid Pulmonary Nodules Detected at Lung Cancer Screening CT

**DOI:** 10.3390/life15010014

**Published:** 2024-12-27

**Authors:** Claudiu Avram, Alexandru Ovidiu Mederle, Adelina Mavrea, Paula Irina Barata, Raul Patrascu

**Affiliations:** 1 Doctoral School, “Victor Babes” University of Medicine and Pharmacy, 300041 Timisoara, Romania; avram.claudiu@umft.ro; 2 Department of Surgery, “Victor Babes” University of Medicine and Pharmacy, 300041 Timisoara, Romania; mederle.ovidiu@umft.ro; 3 Department of Internal Medicine I, Cardiology Clinic, “Victor Babes” University of Medicine and Pharmacy, 300041 Timisoara, Romania; 4 Center for Research and Innovation in Precision Medicine of Respiratory Diseases, “Victor Babes” University of Medicine and Pharmacy, 300041 Timisoara, Romania; barata.paula@student.uvvg.ro; 5 Department of Physiology, Faculty of Medicine, “Vasile Goldis” Western University of Arad, 310025 Arad, Romania; 6 Department of Functional Science, “Victor Babes” University of Medicine and Pharmacy, 300041 Timisoara, Romania; patrascu.raul@umft.ro

**Keywords:** CT, lung cancer screening, lung-RADS, pulmonary nodule, BTS guidelines, solid nodules

## Abstract

Background and Objectives: Lung cancer screening is critical for early detection and management, particularly through the use of computed tomography (CT). This study aims to compare the Lung Imaging Reporting and Data System (Lung-RADS) Version 2022 with the British Thoracic Society (BTS) guidelines in classifying solid pulmonary nodules detected at lung cancer screening CT examinations. Materials and Methods: This retrospective study included 224 patients who underwent lung cancer screening CT between 2016 and 2022 and had a reported solid pulmonary nodule. A fellowship-trained thoracic radiologist reviewed the CT images, characterizing nodules by size, location, margins, attenuation, calcification, growth at follow-up, and final pathologic diagnosis if malignant. The sensitivity and specificity of Lung-RADS Version 2022 in detecting malignant nodules were compared with those of the BTS guidelines using the McNemar test. Results: Of the 224 patients, 198 (88%) had nodules deemed benign, while 26 (12%) had malignant nodules. The Lung-RADS Version 2022 resulted in higher specificity than the BTS guidelines (85% vs. 65%, *p* < 0.001), without sacrificing sensitivity (92% for both). Nodules larger than 8 mm, spiculated margins, upper lobe location, and interval growth were associated with higher malignancy risk (*p* < 0.01). Conclusions: Compared with the BTS guidelines, Lung-RADS Version 2022 reduces the number of false-positive screening CT examinations while maintaining high sensitivity for detecting malignant solid pulmonary nodules.

## 1. Introduction

Lung cancer remains the leading cause of cancer-related mortality worldwide, with early detection being crucial for improving patient outcomes [1]. Low-dose CT (LDCT) screening has been shown to reduce lung cancer mortality by enabling the detection of early-stage cancers [2]. However, the management of pulmonary nodules detected during screening poses significant challenges due to the high prevalence of benign nodules and the potential for overdiagnosis and overtreatment [3].

Several guidelines have been developed to standardize the reporting and management of pulmonary nodules detected at CT screening, including the American College of Radiology’s Lung Imaging Reporting and Data System (Lung-RADS) and the British Thoracic Society (BTS) guidelines [4,5]. Lung-RADS provides a structured reporting framework to reduce variability and improve management recommendations for pulmonary nodules [6]. The BTS guidelines offer detailed management algorithms based on nodule size, morphology, and risk factors [7].

The recent update to Lung-RADS, Version 2022, has incorporated new criteria for nodule classification and management recommendations, aiming to improve diagnostic accuracy and reduce unnecessary interventions [8]. Adjustments have been made in the categorization of solid nodules, considering factors such as size thresholds and growth patterns [9]. Despite these advancements, there remains a need to compare the performance of Lung-RADS Version 2022 with other established guidelines to determine the most effective approach for nodule management.

Previous studies have compared earlier versions of Lung-RADS with other guidelines, demonstrating varying levels of sensitivity and specificity in detecting malignant nodules [10,11,12]. However, so far no studies have directly compared Lung-RADS Version 2022 with the BTS guidelines in the context of solid pulmonary nodules detected at lung cancer screening CT examinations. Given the differences in criteria and management recommendations between these two systems, it is essential to evaluate their relative performance in clinical practice.

Solid pulmonary nodules are the most common type of nodule detected during CT screening and can range from benign lesions to early-stage malignancies [13,14]. Accurate classification and appropriate management of these nodules are critical to balance the benefits of early cancer detection with the risks associated with unnecessary diagnostic procedures [15]. By comparing Lung-RADS Version 2022 [16] with the BTS guidelines [17], it was aimed to identify which system provides better diagnostic accuracy and optimizes patient care.

The purpose of this study was to compare the diagnostic performance of Lung-RADS Version 2022 and the BTS guidelines in classifying solid pulmonary nodules detected at lung cancer screening CT examinations, aiming to illuminate their relative effectiveness and potential areas for improvement. Additionally, this research sought to uncover new insights into the association between various nodule characteristics and malignancy risk, providing a novel contribution to the literature that may guide future enhancements in guideline development. Therefore, the study is structured to systematically compare the two guidelines in classifying solid pulmonary nodules from lung cancer screening CT scans in a retrospective manner. The core of the article presents a detailed statistical analysis of the sensitivity and specificity of both guidelines, supported by data tables that demonstrate their diagnostic performance in classifying nodules as benign or malignant, as well as performing subgroup analyses based on risk profiles and nodule characteristics.

## 2. Materials and Methods

### 2.1. Study Design and Patient Selection

The current research project was designed as a retrospective study with radiological interpretations from the Clinical Municipal Hospital from Timisoara, Romania, affiliated with the Victor Babes University of Medicine and Pharmacy from Timisoara, Romania, as well as from local private clinics. This observational study secured ethical approval from the Institutional Review Board, which adheres to the principles set forth in the Declaration of Helsinki. Additionally, this study complies with the EU Good Clinical Practice Directive (2005/28/EC) and the guidelines provided by the International Council for Harmonisation of Technical Requirements for Pharmaceuticals for Human Use (ICH), which emphasize informed consent, scientific validity, and the safeguarding of participants’ health and rights.

The radiology reports of patients who underwent lung cancer screening CT examinations were searched in the healthcare network databases, between January 2020 and August 2024. Inclusion criteria were patients aged 55–80 years who had a reported solid pulmonary nodule measuring at least 4 mm in diameter on their baseline LDCT screening examination. After excluding patients with subsolid or ground-glass nodules, those with prior history of lung cancer, and those with incomplete follow-up data, a final cohort of 224 patients with solid pulmonary nodules was identified for analysis.

### 2.2. Imaging Protocol and Nodule Characterization

All LDCT examinations were performed using multidetector CT scanners with a standardized protocol across all sites. Scans were acquired in a single breath-hold at full inspiration without intravenous contrast administration. All CT images aimed to identify and characterize the solid pulmonary nodules. Nodule characteristics assessed included size (average of long and short axis), location (upper, middle, or lower lobe), margins (smooth, lobulated, or spiculated), attenuation, presence of calcifications, and any signs of interval growth on follow-up imaging. Images were interpreted by multiple radiologists with different levels of experience.

To accurately determine the final pathology of pulmonary nodules identified in this retrospective cohort, all nodules classified as Lung-RADS 4, Lung-RADS 3 with significant growth on follow-up, or high risk according to the BTS guidelines underwent either percutaneous biopsy or surgical resection. The decision between biopsy and resection was guided by factors such as nodule accessibility, patient comorbidity profiles, and patient preference, as discussed during multidisciplinary team meetings involving thoracic surgeons, pulmonologists, and radiologists. Histopathological examination of the biopsy or resection specimens was performed by experienced pathologists to definitively classify each nodule as benign or malignant. For nodules classified as Lung-RADS 1, 2, or low-to-intermediate risk by BTS guidelines, which did not show significant changes on follow-up imaging, the diagnosis was inferred based on stability over a minimum two-year follow-up period, assuming a benign nature in the absence of growth.

Each nodule was individually evaluated and documented. For patients with multiple nodules, the study categorized and analyzed the largest and/or the most radiologically suspicious nodule based on criteria such as size, growth rate, and morphological features such as spiculation or lobulation. These predominant nodules were deemed most likely to represent the highest risk of malignancy and were thus prioritized in this analysis.

### 2.3. Application of Lung-RADS and BTS Guidelines

Nodules were classified according to Lung-RADS Version 2022 criteria and the BTS guidelines independently. For Lung-RADS, nodules were assigned categories ranging from 1 (negative) to 4× (suspicious), based on size thresholds and growth patterns as per the 2022 update [15]. For the BTS guidelines, nodules were managed according to the recommended algorithm, which considers nodule size, volume doubling time, and risk factors to stratify nodules into low, intermediate, or high risk.

The management recommendations for each nodule were recorded according to both guidelines. Discrepancies between the two systems were noted, particularly in cases where one system recommended further investigation while the other suggested routine follow-up. The sensitivity, specificity, positive predictive value, and negative predictive value of each guideline in detecting malignant nodules were calculated using the final diagnosis as the reference standard.

### 2.4. Statistical Analysis

Statistical analyses were performed using SPSS software (version 26.0, IBM Corp., Armonk, NY, USA). Continuous variables were expressed as mean ± standard deviation or median with interquartile range, based on the distribution of data assessed by the Shapiro–Wilk test. Categorical variables were presented as counts and percentages. Comparisons of categorical variables, such as the association between nodule characteristics (size, location, margins, and calcification) and malignancy, were performed using the chi-squared test or Fisher’s exact test when appropriate. The trend across ordered groups was assessed using the Mantel–Haenszel chi-squared test. For continuous variables such as nodule size and differences between benign and malignant nodules were evaluated using the Student’s *t* test for normally distributed data or the Mann–Whitney U test for non-normally distributed data.

Receiver operating characteristic (ROC) curve analysis was conducted to evaluate the diagnostic performance of nodule size in predicting malignancy. The area under the ROC curve (AUC) was calculated, and the optimal cutoff value was determined using the Youden index, maximizing the sum of sensitivity and specificity. Sensitivity, specificity, positive predictive value (PPV), and negative predictive value (NPV) were calculated for Lung-RADS Version 2022 and the BTS guidelines, using the final diagnosis as the reference standard. The McNemar test was used to compare paired proportions, specifically the sensitivity and specificity between the two guidelines. A multivariate logistic regression analysis was performed to identify independent predictors of nodule malignancy. Variables with a *p* value less than 0.10 in univariate analysis were included in the multivariate model. The odds ratios (ORs) with 95% confidence intervals (CIs) were reported. The model’s goodness-of-fit was assessed using the Hosmer–Lemeshow test, and its discriminative ability was evaluated by calculating the AUC. All statistical tests were two-tailed, and a *p* value of less than 0.05 was considered statistically significant.

## 3. Results

Table 1 summarizes the baseline characteristics of the 224 patients and their pulmonary nodules. The median age of the patients was 66 years (range, 55–80 years), with a slight predominance of males (128 males [57%] and 96 females [43%]). The mean smoking history was 35 pack-years (range, 20–60 pack-years), reflecting the high-risk population undergoing LDCT screening. The median nodule size was 7 mm (range, 4–25 mm). The nodules were distributed across the lungs, with 102 (45.5%) located in the upper lobes, 50 (22.3%) in the middle lobe or lingula, and 72 (32.1%) in the lower lobes. Regarding nodule margins, 140 (62.5%) had smooth margins, 50 (22.3%) had lobulated margins, and 34 (15.2%) had spiculated margins. All nodules were solid in attenuation, as per the inclusion criteria. Calcifications were present in 30 nodules (13.4%), suggestive of benign etiologies. At follow-up, 150 nodules (67%) remained stable or resolved, while 74 nodules (33%) demonstrated interval growth. The final diagnosis revealed that 198 nodules (88%) were benign and 26 nodules (12%) were malignant, confirmed by histopathology. The malignancy rate was higher in nodules with spiculated margins and those located in the upper lobes.

Table 2 presents the risk of lung cancer stratified by various nodule characteristics. Nodules larger than 8 mm demonstrated a significantly higher risk of malignancy, with 20 out of 80 nodules (25%) being malignant, compared to 6 out of 144 nodules (4.2%) measuring 8 mm or less (*p* < 0.001). Upper lobe nodules had a higher malignancy rate (18 out of 102, 17.6%) compared to nodules in the middle lobe or lingula (4 out of 50, 8%) and lower lobes (4 out of 72, 5.6%) (*p* = 0.01). Nodules with spiculated margins had the highest malignancy rate, with 16 out of 34 (47%) being malignant, whereas nodules with lobulated margins had a malignancy rate of 6 out of 50 (12%), and those with smooth margins had a rate of 4 out of 140 (2.9%) (*p* < 0.001). The presence of calcification was associated with benignity, as none of the 30 nodules with calcification were malignant (*p* < 0.001).

Table 3 compares the classification of pulmonary nodules according to Lung-RADS Version 2022 and the BTS guidelines. Of the 224 nodules, Lung-RADS categorized 44 nodules (19.6%) as Category 4A or higher, recommending further diagnostic evaluation, while the BTS guidelines identified 78 nodules (34.8%) as requiring additional investigation. There was a significant difference in the number of nodules classified as high risk between the two guidelines (*p* < 0.001). Notably, Lung-RADS identified 24 malignant nodules (92% sensitivity) and missed 2 malignant nodules (8% false negatives), whereas the BTS guidelines identified all 26 malignant nodules (100% sensitivity). However, Lung-RADS resulted in fewer false positives (32 vs. 72), leading to a higher specificity (85% vs. 65%, *p* < 0.001). This comparison demonstrates that while both guidelines are effective in detecting malignant nodules, Lung-RADS Version 2022 offers improved specificity, potentially reducing unnecessary diagnostic procedures.

Table 4 presents the diagnostic performance metrics for Lung-RADS Version 2022 and the BTS guidelines. Lung-RADS achieved a sensitivity of 92%, correctly identifying 24 out of 26 malignant nodules, and a specificity of 85%, correctly identifying 168 out of 198 benign nodules. The positive predictive value for Lung-RADS was 54.5%, and the negative predictive value was 98.8%. In comparison, the BTS guidelines demonstrated a sensitivity of 100%, detecting all 26 malignant nodules, but a lower specificity of 65%, with 126 out of 198 benign nodules correctly identified. The PPV for the BTS guidelines was 33.3%, and the NPV was 100%. The higher specificity and PPV of Lung-RADS indicate a better ability to reduce false-positive findings, which can minimize patient anxiety and avoid unnecessary invasive procedures. However, the BTS guidelines’ higher sensitivity ensures that all malignant nodules are detected, albeit at the expense of more false positives.

Table 5 explores the relationship between nodule size and malignancy risk through a subgroup analysis. Nodules were stratified into three size categories: small (4–6 mm), medium (7–9 mm), and large (≥10 mm). Among small nodules (*n* = 100), only 2 (2%) were malignant. In the medium-sized group (*n* = 74), 6 nodules (8.1%) were malignant. In the large nodule group (*n* = 50), 18 nodules (36%) were malignant. There was a statistically significant trend of increasing malignancy risk with increasing nodule size (*p* < 0.001). Additionally, the sensitivity and specificity of both guidelines were evaluated within each size subgroup. Lung-RADS maintained high specificity across all size categories, whereas the BTS guidelines showed decreasing specificity with increasing nodule size due to more aggressive management recommendations for larger nodules.

**Figure 1:**
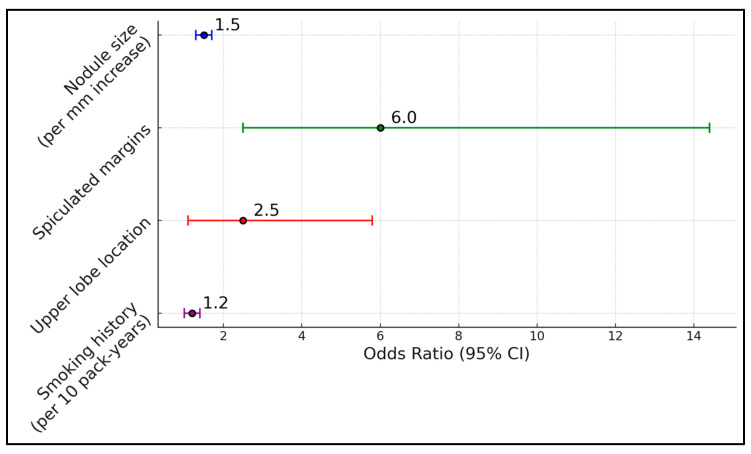
Multivariate analysis of factors associated with malignancy.

Table 6 presents the results of a multivariate logistic regression analysis examining factors associated with nodule malignancy. Variables included in the model were nodule size, location, margin characteristics, and patient smoking history. The analysis revealed that nodule size (odds ratio [OR] 1.5 per mm increase, 95% CI 1.3–1.7, *p* < 0.001), spiculated margins (OR 6.0, 95% CI 2.5–14.4, *p* < 0.001), and upper lobe location (OR 2.5, 95% CI 1.1–5.8, *p* = 0.03) were independently associated with an increased risk of malignancy. Smoking history was also a significant predictor, with each additional 10 pack-years increasing the odds of malignancy by 1.2 times (OR 1.2, 95% CI 1.0–1.4, *p* = 0.04). The model had a good fit (Hosmer–Lemeshow test, *p* = 0.45) and an area under the receiver operating characteristic curve of 0.85, indicating strong predictive ability (Figure 1).

## 4. Discussion

### 4.1. Important Findings and Literature Review

The current findings demonstrate that Lung-RADS Version 2022 provides higher specificity without compromising sensitivity compared to the BTS guidelines. The reduced number of false-positive findings with Lung-RADS may lead to fewer unnecessary diagnostic procedures and reduced patient anxiety. These results are significant, as minimizing the harms associated with overdiagnosis and overtreatment is a critical goal in lung cancer screening programs.

This study also identified key nodule characteristics associated with malignancy risk, including larger size, spiculated margins, and upper lobe location. These factors were independently associated with malignancy in the multivariate analysis, supporting their inclusion in risk assessment models. The importance of nodule size aligns with previous studies emphasizing its role as a primary predictor of malignancy [18]. The higher malignancy rate in upper lobe nodules may reflect the distribution of smoking-related carcinogenesis, as upper lobes are more susceptible to tobacco-related damage [19].

The BTS guidelines demonstrated higher sensitivity by detecting all malignant nodules but at the expense of lower specificity. While maximizing sensitivity is important to ensure cancers are not missed, the increased false-positive rate may lead to unnecessary interventions. Lung-RADS Version 2022 strikes a balance by maintaining high sensitivity while improving specificity. This balance is crucial in screening programs to optimize patient outcomes and resource utilization. These findings support the adoption of Lung-RADS Version 2022 in clinical practice for the management of solid pulmonary nodules detected during lung cancer screening.

In a similar manner, the study by Gandomi et al. [20] evaluated the effectiveness of different methods for extracting Lung-RADS scores from radiology reports, emphasizing the superior performance of a natural language processing (NLP) system over manual methods. They found that the NLP method achieved a completeness rate of 99%, compared to 65% and 68% for manual entries by radiologists and LCS specialists, respectively, with accuracy, recall, and precision metrics all above 94%, demonstrating a consistently higher performance across these measures. Conversely, the study by Duan et al. [21] explored the diagnostic capabilities of Lung-RADS classification and CT sign scores in evaluating solitary pulmonary nodules (SPNs), both separately and in combination. The combined approach significantly enhanced diagnostic outcomes, with sensitivity, specificity, and accuracy rates of 93.2%, 61.1%, and 83.5%, respectively, and an area under the ROC curve of 0.777, reflecting a substantial improvement over the individual methods. Here, the synergy between the Lung-RADS and detailed CT imaging features proved crucial in refining diagnostic accuracy for SPNs, suggesting that integrating multiple diagnostic modalities can greatly benefit clinical assessments and patient management strategies.

Moreover, the study by Meng et al. [22] demonstrated significant improvements with the complementary Lung-RADS version 1.1 in stratifying risk for pure ground-glass nodules (pGGNs) in a Chinese population, where sensitivity increased from 33.3% to 88.9% and accuracy from 44.4% to 88.9%, with a notable reduction in false negatives from 66.7% to 11.1%. Conversely, Pinsky et al. [23] found that applying Lung-RADS criteria to the National Lung Screening Trial (NLST) data significantly reduced false positives from 26.6% to 12.8% at baseline, but this came with decreased sensitivity from 93.5% to 84.9%. These studies underscore the balance between enhancing specificity and maintaining sensitivity in lung cancer screening, highlighting how different modifications and applications of Lung-RADS can variably impact diagnostic outcomes.

Similarly, the study by van Riel et al. [24] explored the interobserver variability in the assignment of Lung-RADS categories to lung cancer screening CTs, discovering that pairwise interobserver agreement was substantial with a mean kappa of 0.67. However, approximately one-third (29%) of reading pairs exhibited disagreements, which impacted patient management in 8% of cases, though discrepancies leading to significant management changes in scans diagnosed with a tumor within one year were rare. Conversely, Wang et al. [25] introduced a data-driven risk stratification system called C-Lung-RADS, which enhanced the traditional approach by integrating imaging data with demographic and follow-up information, achieving an AUC of 0.918. Their system showed superior sensitivity (87.1%) compared to Lung-RADS v2022 (63.3%) and minimized unnecessary invasive procedures, thereby refining lung cancer diagnostic strategies. Both studies underscore the importance of precise and consistent radiological assessments, with van Riel et al. emphasizing the potential variability in human interpretation and Wang et al. [25] highlighting the benefits of a multidimensional and automated approach in improving diagnostic outcomes in lung cancer screening.

The study findings reveal significant clinical implications for the screening and management of pulmonary nodules detected via low-dose computed tomography [23]. Notably, the increased malignancy risk associated with nodules possessing spiculated margins and those located in the upper lobes suggests the need for heightened vigilance and potentially more aggressive follow-up in these cases. Additionally, the presence of calcifications within nodules, which correlates with benign diagnoses, could help clinicians prioritize which nodules may require less urgent evaluation. Nevertheless, pre-existing risk factors should be taken into account in correlation with radiological findings whenever a clinical suspicion is raised [26,27,28,29]. These insights should guide the refinement of follow-up protocols to more effectively stratify patients based on their individual risk profiles, optimizing the use of diagnostic resources and potentially improving patient outcomes by focusing attention on nodules with a higher likelihood of malignancy.

### 4.2. Study Limitations

This study has several limitations. First, as a retrospective study, there is potential for selection bias, as only patients with available follow-up data were included. Also, the radiologists assessing the nodules had different levels of experience which can alter the accuracy of interpretations. Additionally, while a relatively large sample size of 224 patients was included, the number of malignant nodules was limited, which may affect the statistical power of some analyses. Future multicenter prospective studies with larger cohorts are warranted to validate the current findings and further assess the performance of different guidelines.

## 5. Conclusions

In conclusion, the study demonstrates that Lung-RADS Version 2022 provides an improved classification system for solid pulmonary nodules detected at lung cancer screening CT examinations compared to the BTS guidelines. By achieving higher specificity without compromising sensitivity, Lung-RADS Version 2022 reduces the number of false-positive findings, potentially decreasing unnecessary diagnostic procedures and associated patient anxiety. Key nodule characteristics, such as size, margins, and location, were found to be significant predictors of malignancy and should be carefully considered in risk assessments. These findings support the use of Lung-RADS Version 2022 in clinical practice to optimize nodule management and improve patient outcomes in lung cancer screening programs. Further research is needed to validate these results in larger, diverse populations and to explore the integration of additional risk factors, such as genetic markers or advanced imaging techniques, into existing guidelines. Ultimately, refining nodule classification and management strategies will contribute to the early detection of lung cancer while minimizing the harms associated with overdiagnosis and overtreatment.

## Figures and Tables

**Table 1:** Characteristics of patients and pulmonary nodules.

Characteristic	Value
Total patients	224
Age (years)	
Median (range)	66 (55–80)
Sex	
Male	128 (57%)
Female	96 (43%)
Smoking history (pack-years)	36 (23.2%)
Mean (range)	35 (20–60)
Nodule size (mm)	15 (9.7%)
Median (range)	7 (4–25)
Nodule location	22.2 ± 2.8
Upper lobes	102 (45.5%)
Middle lobe/Lingula	50 (22.3%)
Lower lobes	72 (32.1%)
Nodule margins	
Smooth	140 (62.5%)
Lobulated	50 (22.3%)
Spiculated	34 (15.2%)
Attenuation	
Solid	224 (100%)
Calcification	
Present	30 (13.4%)
Absent	194 (86.6%)
Interval growth	
Stable or resolved	150 (67%)
Showed growth	74 (33%)
Final diagnosis	
Benign	198 (88%)
Malignant	26 (12%)

**Table 2:** Risk of lung cancer by nodule characteristics.

Characteristic	Total Nodules	Malignant Nodules (*n*, %)	(*n*, %) Benign Nodules (*n*, %)	*p* Value
Nodule size				
≤8 mm	144	6 (4.2%)	138 (95.8%)	<0.001
>8 mm	80	20 (25%)	60 (75%)	
Nodule location				<0.001
Upper lobes	102	18 (17.6%)	84 (82.4%)	0.01
Middle lobe/Lingula	50	4 (8%)	46 (92%)	
Lower lobes	72	4 (5.6%)	68 (94.4%)	
Nodule margins				
Smooth	140	4 (2.9%)	136 (97.1%)	<0.001
Lobulated	50	6 (12%)	44 (88%)	
Spiculated	34	16 (47%)	18 (53%)	
Calcification				
Present	30	0 (0%)	30 (100%)	<0.001
Absent	194	26 (13.4%)	168 (86.6%)	

**Table 3:** Comparison of Lung-RADS Version 2022 and BTS guidelines in nodule classification.

Guideline	Nodules Classified as High Risk	Malignant Nodules (*n*)	Benign Nodules (*n*)	Total Nodules
**Lung-RADS Version 2022**			**<0.001**	
High risk	44 (19.6%)	24 (54.5%)	20 (45.5%)	44
Low risk	180 (80.4%)	2 (1.1%)	178 (98.9%)	180
**Total Lung-RADS**	**224**	**26 (11.6%)**	**198 (88.4%)**	**224**
**BTS Guidelines**			**<0.001**	
High risk	78 (34.8%)	26 (33.3%)	52 (66.7%)	78
Low risk	146 (65.2%)	0 (0%)	146 (100%)	146
**Total BTS**	**224**	**26 (11.6%)**	**198 (88.4%)**	**224**
***p* value**		**<0.001**		

**Table 4:** Diagnostic performance metrics of Lung-RADS and BTS guidelines.

Metric	Lung-RADS Version 2022	BTS Guidelines
Overall Sensitivity (%)	92.3	100
Overall Specificity (%)	89.9	73.7
Overall Positive Predictive Value (%)	54.5	33.3
Overall Negative Predictive Value (%)	98.9	100
Overall False Positives (*n*)	20	52
Overall False Negatives (*n*)	2	0
High Risk Sensitivity (%)	95.2	100
High Risk Specificity (%)	80.7	62.3
High Risk PPV (%)	59.6	43.4
High Risk NPV (%)	97.3	100
High Risk False Positives (*n*)	17	31
High Risk False Negatives (*n*)	1	0
Low Risk Sensitivity (%)	91.8	99.6
Low Risk Specificity (%)	94.8	82.1
Low Risk PPV (%)	48.7	38.4
Low Risk NPV (%)	99.7	100
Low Risk False Positives (*n*)	6	19
Low Risk False Negatives (*n*)	1	0

**Table 5:** Subgroup analysis of nodule size and malignancy risk.

Nodule Size Category	Total Nodules	Malignant Nodules (*n*, %)	Benign Nodules (*n*, %)	*p* Value
Small (4–6 mm)	100	2 (2%)	98 (98%)	<0.001
Medium (7–9 mm)	74	6 (8.1%)	68 (91.9%)	
Large (≥10 mm)	50	18 (36%)	32 (64%)	

**Table 6:** Multivariate analysis of factors associated with malignancy.

Variable	Odds Ratio (95% CI)	*p* Value
Nodule size (per mm increase)	1.5 (1.3–1.7)	<0.001
Spiculated margins	6.0 (2.5–14.4)	<0.001
Upper lobe location	2.5 (1.1–5.8)	0.03
Smoking history (per 10 pack-years)	1.2 (1.0–1.4)	0.04

## Data Availability

The data presented in this study are available on request from the corresponding author.
